# Optimized Sedation Improves Colonoscopy Quality Long-Term

**DOI:** 10.1155/2015/195093

**Published:** 2015-01-08

**Authors:** Konstantinos Triantafyllou, Athanasios D. Sioulas, Theodora Kalli, Nikolaos Misailidis, Dimitrios Polymeros, Ioannis S. Papanikolaou, George Karamanolis, Spiros D. Ladas

**Affiliations:** ^1^Hepatogastroenterology Unit, Second Department of Internal Medicine and Research Institute, Attikon University General Hospital, Medical School, Athens University, 12462 Haidari, Greece; ^2^Academic Department of Gastroenterology, Laiko General Hospital, Medical School, Athens University, 11527 Athens, Greece

## Abstract

*Background*. Quality monitoring and improvement is prerequisite for efficient colonoscopy. *Aim*. To assess the effects of increased sedation administration on colonoscopy performance. *Materials and Methods*. During Era 1 we prospectively measured four colonoscopy quality indicators: sedation administration, colonoscopy completion rate, adenoma detection rate, and early complications rate in three cohorts: cohort A: intention for total colonoscopy cases; cohort B: cohort A excluding bowel obstruction cases; cohort C: CRC screening-surveillance cases within cohort B. We identified deficiencies and implemented our plan to optimize sedation. We prospectively evaluated its effects in both short- (Era 2) and long-term period (Era 3). *Results*. We identified that sedation administration and colonoscopy completion rates were below recommended standards. After sedation optimization its use rate increased significantly (38.1% to 55.8% to 69.5%) and colonoscopy completion rate increased from 88.3% to 90.6% to 96.4% in cohort B and from 93.2% to 95.3% to 98.3% in cohort C, in Eras 1, 2, and 3, respectively. Adenoma detection rate increased in cohort C (25.9% to 30.6% to 35%) and early complications rate decreased from 3.4% to 1.9% to 0.3%. Most endoscopists increased significantly their completion rate and this was preserved long-term. *Conclusion*. Increased sedation administration results in long-lasting improvement of colonoscopy quality indicators.

## 1. Introduction

Colonoscopy has been largely accepted as an effective tool for colorectal cancer (CRC) screening, given its ability to detect and remove identified polyps [1]. By achieving interruption in adenoma-carcinoma sequence, increased colonoscopy utilization is possibly associated with the recently observed decline in the incidence of CRC and its diagnosis at earlier stages [2, 3].

Despite the evidence supporting the effectiveness of colonoscopy, emerging data underline its imperfections. Adenoma miss rates have been estimated to reach 24% in tandem colonoscopy studies; proximal colon seems to be less protected compared to distal colon and interval cancers development is not as rare as believed [4–6]. Numerous technical-, patient-, and endoscopist-related factors have been studied to explain variability in colonoscopy outcomes affecting the overall quality of the procedure [7–9].

In this setting, international medical associations such as European Society of Gastrointestinal Endoscopy (ESGE), the American College of Gastroenterology (ACG), and the American Society for Gastrointestinal Endoscopy (ASGE) have proposed several quality indicators to establish competence in colonoscopy as well as to define areas for continuous quality improvement (CQI) [10, 11].

As literature discloses, plenty of interventions have been implemented aiming at maximizing the quality of colonoscopy and substantially decreasing CRC rates. These include various CQI programs incorporating changes in procedural, technical, and/or physician-related parameters; nevertheless, a diversity of effects, as judged by established quality indicators, has been produced [12–15]. Additionally, data regarding the long-term results of such interventions are lacking, deterring their over-time sustainability assessment.

Taking the above into account, our primary objective was to determine both the short- and long-term effects of increased sedation administration on the quality of colonoscopies performed at an academic endoscopy facility. Secondarily, we evaluated individual endoscopists' performance changes over time related to sedation administration optimization.

## 2. Materials and Methods

### 2.1. Design and Definitions

In our endoscopy facility, we prospectively measured four colonoscopy quality indicators, namely, (i) sedation administration rate (SAR): proportion of colonoscopies with intravenous sedation-analgesia administration; (ii) colonoscopy completion rate (CCR): proportion of colonoscopies where caecum or terminal ileum was intubated or the anastomosis after resection was reached; (iii) adenoma detection rate (ADR): proportion of colonoscopies with at least one histologically confirmed adenoma; and (iv) early complication rate (CR): proportion of colonoscopies associated with complications occurring during colonoscopy or until discharge.

We measured these quality indicators in three predefined patients' cohorts: cohort A: cases with intention for total colonoscopy; cohort B: cohort A excluding cases with bowel obstruction; and cohort C: CRC screening-surveillance cases within cohort B. Assigning subjects in these cohorts allowed separate investigations of specific outcomes in each group. More precisely, in cohort A, we assessed reasons for incomplete colonoscopy, SAR, and CR; in cohort B we monitored SAR, CCR, and CR; and in cohort C we measured SAR, CCR, ADR, and CR.

**Figure 1:**
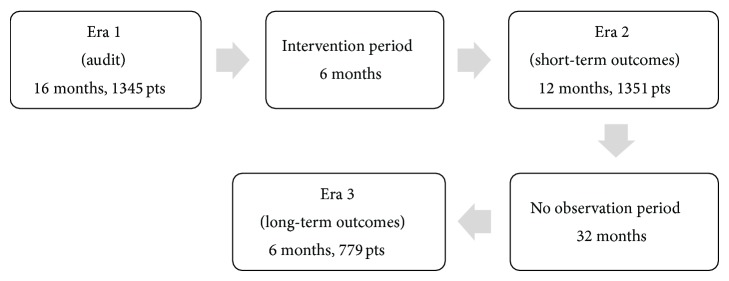
Flow diagram of the study: Eras 1, 2, and 3 of the study are separated by the intervention and nonobservation periods, respectively.

Analyses were performed during three predefined time periods: (1) an initial 16-month audit period (Era 1) from January 2007 to April 2008, when we prospectively collected the baseline measurements of the quality indicators of colonoscopy performance, followed by a 6-month period from May 2008 to October 2008, where deficiencies were identified and the corrective plan was implemented; (2) a subsequent 12-month period (Era 2) from November 2008 to October 2009, where we prospectively assessed the same quality indicators to evaluate the effects of the program on colonoscopy performance; (3) a distant 6-month period (Era 3) from July 2012 to December 2012, to evaluate the durability of the effects of the intervention (Figure 1).

Participating endoscopists were unaware of being monitored during Era 3, in contrast to the previous Eras. In addition, the overall performance of the facility was formally presented and discussed within the staff members at the end of the internal audit, whereas individual results were disclosed by the facility's head to each participating endoscopist in private.

Given the findings of the initial audit period we implemented our action plan involving increased delivery of sedation and analgesia with midazolam and pethidine, respectively. The background of this intervention was published guidelines recommending routine use of sedation in at least 90% of screening colonoscopies [16]. More precisely, specific orders were given to endoscopists in order to perform procedures under conscious sedation, unless contraindicated. Contraindications included patient's unwillingness, known drug allergy and exclusion after ASA grade, comorbidities, and baseline vital signs assessment. The optimized sedation administration schedule consisted of (i) intravenous administration of 1.5 mg of midazolam and 12.5 mg of pethidine at colonoscopy initiation; (ii) medications' dose titration during colonoscopies by 0.5 mg of midazolam and 4 mg of pethidine depending on patient's discomfort and vital signs, up to 6 mg and 100 mg, respectively. Of note, there was an approximately 20% dose reduction in the elderly [17].

### 2.2. Population and Procedures

We measured the colonoscopy quality indicators in all procedures that intended to visualize the whole colon at the Endoscopy Facility of Attikon University General Hospital in Athens, Greece, during the predefined periods.

Colonoscopies were performed on a daily basis, by or under the supervision of rotating senior gastroenterologists. Trainee participation routinely but not unanimously occurred. Participating trainees started the examination and proceeded until no progression could be achieved and senior gastroenterologists took over.

Procedures were performed by using Olympus CF-Q145L standard-definition white-light colonoscopes (Olympus Corporation, Tokyo, Japan) after bowel preparation with either 4 L of polyethylene glycol or 90 mL of sodium phosphate. Bowel preparation quality was characterized as adequate (excellent/good) or inadequate (fair/poor) for the right (caecum, ascending, and transverse) and the left (descending, sigmoid, and rectum) colon, separately [18].

During the examinations, pulse rate, arterial blood pressure, oxygen saturation, and level of consciousness were being monitored. Supplemental oxygen was routinely delivered via nasal catheters. Intravenous conscious sedation and analgesia was administered on demand during the audit period and according to the corrective plan during the other two periods. Reversal agents (flumazenil, naloxone) were used in case of sedation-related complications.

For each eligible for analysis procedure the following data were collected: endoscopist identification; participation of a trainee; patient characteristics (age, gender, ASA grade, in- or outpatient); indication for colonoscopy; sedation and oxygen administration; bowel preparation quality; cecal intubation (or visualization of the anastomosis, as appropriate) and the reason that explained failure, if it occurred; adenoma detection and early (e.g., occurring during examination or until discharge) complication occurrence and type. All data regarding patients' characteristics, procedures, and quality parameters were captured in a standardized report card.

### 2.3. Ethical Considerations

Institutional ethics committee approval for our audit was not needed, given that our CQI program was regarded to be a service evaluation in order to provide our patients with best care. No specific informed consent was obtained, since all patients received the standard-of-care without reference to any study. However, all patients signed standard informed consent for colonoscopy. Additionally, data were deidentified before analysis and their collection and review were considered as part of the internal evaluation.

### 2.4. Statistical Analysis

Continuous variables are presented as means or medians and standard deviations. Binary variables are reported as percentages with corresponding 95% confidence intervals (CI). Variables are presented separately in each Era in relation to patients' cohorts and endoscopists. Nonparametric tests were used to detect differences, as appropriate. A *P* value less than 0.05 indicated a statistical significance.

## 3. Results

We evaluated data from 1345 (53% male), 1351 (51.1% male), and 779 (52.2% male) patients with median ages (±SD) of 64 (12), 65 (14.7), and 63 (15.2) years during Eras 1, 2, and 3, respectively. Throughout the assessment the majority of colonoscopies were diagnostic (70.6%, 68.3%, and 62.1% during Eras 1, 2, and 3, resp.) and the most common indication was “screening-surveillance.” Regarding trainee participation, a significant increase was observed only between Eras 2 and 3 (57.6% versus 73.1%; *P* < 0.0001), but not between Eras 1 and 2 (56.1% versus 57.6%; *P* = 0.46). After excluding cases with indication for partial colon examination, cases were assigned to the three cohorts for further evaluation.

As shown in Table 1, during the audit period (Era 1), we identified a low (41.5%) cohort A SAR, whereas CCR was below the recommended standards in cohorts B and C (88.3% and 93.2%, resp.). Reasons for colonoscopy failures, as best assessed in cohort A at Era 1, were patient's intolerance (30%), obstruction of the bowel lumen (25%), inadequate preparation (19%), acute colonic angulations or fixed loops (19%), and early complications (9%). Therefore, we hypothesized that increasing sedation and analgesia administration, as described above, could improve patient comfort and affect positively the overall performance of colonoscopy.

### 3.1. Primary Endpoint Outcomes

After implementation of the corrective plan, SAR increased significantly in cohort A, both between Eras 1 and 2 (41.5% versus 63.5%; *P* < 0.0001) and Eras 2 and 3 (63.5% versus 86.5%; *P* < 0.0001). This increase was uniformly distributed to patients' cohorts B and C, as shown in Table 1. As a consequence, during Era 2, there was a significant difference regarding the reasons for incomplete colonoscopy since the percentage of intolerance and of complication related cases dramatically fell (16% and 4.5%, resp.).

CCR in cohorts B and C exhibited constant improvement through the successive Eras. In cohort B significant changes were noted between Eras 2 and 3 (90.6% versus 96.4%; *P* < 0.0001) and between Eras 1 and 3 (88.3 versus 96.4%; *P* < 0.0001), respectively. In cohort C, statistical significance was detected between Eras 1 and 3 (93.2% versus 98.3%; *P* = 0.005).

ADR increased constantly in cohort C (from 25.9% to 30.6% to 35% in Eras 1, 2, and 3, resp.); however, significance has been achieved only in Era 3 compared to Era 1 (*P* = 0.018).

CR steadily decreased significantly in cohort A during study time frames (3.4% versus 1.9%; *P* = 0.025 and 1.9% versus 0.3%; *P* = 0.005, between Eras 1 and 2 and Eras 2 and 3, resp.). This decrease was uniformly detected in cohorts B and C, although statistical significance was detected only in cohort B (Table 1). There were 44, 22, and two procedure-related early complications during successive Eras. The majority consisted of cardiopulmonary events that were reversed spontaneously or with ventilation and pharmacological interventions. There were two perforations treated surgically during Era 1 compared to no perforation during Era 2 and one conservatively managed during Era 3. With respect to bleeding events, seven, seven, and one were noticed during Eras 1, 2, and 3, respectively, and seven of them required hospitalization. No death occurred.

Trainee involvement had no significant effect on the colonoscopy quality indicators during the three observation periods.

### 3.2. Secondary Endpoints Outcomes

The number and identity of senior endoscopists did not remain constant among different Eras, since some of them left while others entered the practice over time. More precisely, colonoscopic procedures during Era 1 were performed by five senior endoscopists (1, 2, 3, 4, and 5). During the next Era, two of them (1 and 2) had already moved from the hospital, while two others (6 and 7) had been hired. Eventually, during Era 3 three remaining endoscopists (3, 4, and 6) participated in the evaluations. Of note, the number of procedures performed by endoscopist showed a great variability during the three Eras. However, the majority of them performed or supervised more than 200 colonoscopies yearly.

Variability among endoscopists regarding the four quality indicators during the study periods was also evident. SAR exhibited significant variability in almost all Eras. Additionally, CCR and ADR were significantly variable during Era 2. This could be explained by the fact that one newcomer endoscopist performed significantly worse than the rest. As expected, these differences disappeared during Era 3, when endoscopist 7 had left the practice. No significant variability among the endoscopists in terms of CR was observed throughout the evaluations.

When the results were examined by endoscopists, detailed data regarding their performance are presented in Tables 2 and 3. All endoscopists that participated in at least two consecutive evaluations showed significantly increased SAR and improvement regarding CCR (in cohorts B and C) and ADR (in cohort C); however significance was not achieved always. Individual CR showed a decreasing trend throughout the evaluations.

Only two of the endoscopists participated in all three Eras' evaluations. Although there was significant variability among them regarding SAR, they both improved their performance regarding the remaining three quality indicators by achieving a CCR of 95.3%–99.1% during Era 2 and 98.8%–100% during Era 3 and an ADR of 36.5%–39.1% during Era 2 and 38%–42.4% during Era 3. No complications attributed to these two endoscopists were noted in Era 3 (Table 3).

## 4. Discussion

It is well established that variations in colonoscopy quality reflect differences in numerous patient-, procedure-, and endoscopist-related parameters. Taking that into consideration, a great body of interventions has been conducted aiming at enhancing colonoscopy performance and decreasing its native imperfections. Quality improvement programs include internal audits and feedback to individual endoscopists, education in quality indicators, implementation of mandatory withdrawal times, bowel preparation modifications, discussion with poor-performers, introduction in emerging technologies, routine sedation administration, repeat attempts for cecal intubation, report card utilization, and even financial penalties [12, 13, 19–21]. Hence, although several studies show improvement in the quality metrics, uncertainty still exists regarding the long-term effects of these interventions.

As presented, the implementation of increased sedation delivery in the everyday practice improved colonoscopy outcomes. More detailed, significantly increased SAR during the successive Eras was accompanied by subsequent increases in both CCR and ADR in cohorts B and C which exceeded those indicated by international authorities [10, 11] and reached the recently proposed standards [22]. Accordingly, CR constantly decreased throughout the studied periods.

Improved individual endoscopists performance as assessed by the four quality indicators was also evident. Importantly, our results showed that improvement in the quality metrics was long-lasting and applied to both overall service and individual performance.

Our intervention was associated with improved CCR. This result is in keeping with those of previously published data [20, 23]. However, a recent study indicates the exact reverse: higher CCR was observed among endoscopists using less sedation [24]. A possible explanation for this discrepancy could be that the researchers used the unadjusted CCR, instead of that being adjusted for obstruction, as we did in our analyses.

Delivering more sedation and analgesia also led to consistent improvement in ADR over the study period, as our results suggest. Similarly, changes in sedation practices as part of another CQI program increased polyp detection rate (PDR) [20]. However, no data regarding ADR was presented, although PDR and ADR seem to correlate well, at least in segments proximal to the splenic flexure [25]. In contrast, Paspatis et al. demonstrated no difference in PDR and ADR using moderate or deep sedation [26]. Accordingly, a study by Bannert et al. showed no association between sedation and PDR and ADR, while a randomized-controlled observational study did not include sedation among the modifiable factors related to the ADR [23, 27].

It is noteworthy that increased SAR still remained lower than that announced from a nationwide Greek survey (78%) [28], as well as the requirement to administer sedation-analgesia in at least 90% of the patients undergoing screening colonoscopy [16]. This finding might at least partially explain the inability to further decrease the rate of incomplete cases due to patient discomfort in Era 3 and indicates further interpretation. Despite increased SAR, early complication rate continuously decreased, being within the recommended standards. Similarly, a recent study did not correlate sedation administration and early perforation [28], although another one linked additional sedation to delayed postpolypectomy bleeding [29, 30]. Cardiopulmonary reactions accounted for the majority of our early adverse events, in contrast to the study by Paspatis et al. reporting postpolypectomy bleeding as the principal complication. Variation in definitions of bleeding and cardiorespiratory events severity along with recording feasibility might explain the difference [31].

As shown, our plan resulted in improvements, some of significance, in individual endoscopist performance. Almost all participating endoscopists reached and even exceeded the recommended targets for CCR and ADR, with extremely low CR. Variations among them during Era 2 were attributable to the inclusion of a lower performer. The observed improvements are consistent with those reported in older studies evaluating the effects of various interventions [20, 21, 32]. In contrast, Ball et al. chose to concentrate the performance of colonoscopies to the more proficient physicians, as a means to improve quality indicators [33]. It could be stated that better individual performance might have been secondary to the experience acquired over time. However, experience is not a synonym of improvement in colonoscopy. To note, no senior endoscopist was on the learning curve, since each of them had already performed more than 2000 colonoscopies. Interestingly, a Hawthorne effect (changes in behavior due to knowledge of being observed) might have influenced the results. Initial internal audit might have led to additional changes in individual colonoscopy practice, besides sedation's increase, as recommended. These possibly included prolonged withdrawal time, more careful inspection, and retroflexion in rectum and/or cecum. Such changes, although not systematically assessed in this project, underlie the importance of audit in colonoscopy performance improvement.

Trainee participation did not significantly influence colonoscopy quality, as our results suggest. This is in line with a recent meta-analysis showing no difference in both PDR and ADR by fellow involvement during the procedure [34], although other large retrospective studies indicate the opposite [35, 36]. Moreover, we did not detect any significant association between trainee participation and CR, which is in keeping with the reports of other investigators [37, 38]. A reasonable explanation is that trainees, being inexperienced, are usually more cautious and participate in less demanding cases. In a similar manner, trainees did not influence CCR, since senior endoscopists took over in case of persisting difficulty.

The core strength of our results is the evaluation of our intervention's effects not only shortly but also long after its implementation. As presented, improvement in the quality indicators was durable over a 4-year time period following our intervention. Additionally, the endoscopists remained unaware of being monitored during the distant period, highlighting the incorporated beneficial change in their everyday practice. This sustained effectiveness is, as today, unique in the published literature, given that other attempts discuss significant shorter-term effects [20, 21]. Additive strengths of our observation include the prospective design, the quality performance analysis both per unit and per endoscopist, and the low cost of our intervention.

Nevertheless, our evaluation bears several limitations. The first one is the relatively small number of procedures and participating endoscopists, as well as their changes over time. These reflect institutional endogenous weaknesses limiting endoscopy facility staff availability. Second, the single-center setting may underscore the power and generalizability of the results. Third, the fact that propofol administration is not permitted to nonanesthetists physicians in Greece and the availability of anesthesiology service for endoscopic procedures is limited in our hospitals might also limit the generalizability of our observations. Finally, we did not collect data regarding adenoma size and location, serrated lesions, withdrawal times, and patient feedback.

In conclusion, based on the results of an internal audit, our intervention for optimized sedation administration improved colonoscopy quality indicators and individual endoscopist performance. Most importantly, positive effects demonstrated sustainability over a long time period. However, future work is definitely needed to systematically identify barriers and develop specific interventions aiming at enhancing colonoscopy effectiveness in the prevention of CRC.

## Figures and Tables

**Table 1:** Improvement of the four quality indicators after the implementation of the continuous quality improvement program.

Patients cohorts *N* per Era: 1, 2, and 3	SAR, % (CI)			CCR, % (CI)			ADR, % (CI)			CR, % (CI)		
	Era 1	Era 2	Era 3	Era 1	Era 2	Era 3	Era 1	Era 2	Era 3	Era 1	Era 2	Era 3
(A) *N* = 1213,1159,617	41.5 (38.7–44.3)	63.5^a^ (60.7–66.3)	86.5^b,c^ (83.8–89.2)							3.4 (2.4–4.4)	1.9^a^ (1.1–2.7)	0.3^b,c^ (0–0.7)

(B) *N* = 1167,1107,604	42 (39.2–44.8)	65.2^a^ (62.4–68)	87.6^b,c^ (85–90.2)	88.3 (86.5–90.1)	90.6 (88.9–92.3)	96.4^b,c^ (94.9–97.9)				3.4 (2.4–4.4)	2 (1.2–2.8)	0.3^b,c^ (0–0.7)

(C) *N* = 324,402,234	41 (35.6–46.4)	68.7^a^ (64.2–73.2)	88.5^b,c^ (84.4–92.6)	93.2 (90.0–95.9)	95.3 (93.2–97.4)	98.3^c^ (96.6–100)	25.9 (21.1–30.7)	30.6 (26.1–35.1)	35.0^c^ (28.9–41.1)	2.5 (0.8–4.2)	1.5 (0.3–2.7)	0.9 (0–2.1)

*N*: number of colonoscopies; SAR: sedation administration rate; CI: confidence interval; CCR: colonoscopy completion rate; ADR: adenoma detection rate; CR: early complications rate; ^a^significance over Era 1; ^b^significance over Era 2; ^c^significance over Era 1 during Era 3.

**Table 2:** Individual endoscopist performance in cohort B.

Endoscopist *N* per Era: 1, 2, and 3	SAR, % (CI)			CCR, % (CI)			CR, % (CI)		
	Era 1	Era 2	Era 3	Era 1	Era 2	Era 3	Era 1	Era 2	Era 3
(1) *N* = 70,0, 0	30.0 (19.3–40.7)			84.3 (75.8–92.8)			4.3 (0–9.1)		

(2) *N* = 378,0, 0	41.0 (36–46)			88.4 (85–92)			4.2 (2–6)		

(3) *N* = 208,204,146	38.5 (32–45)	60.3^a^ (53–67)	77.4^b,c^ (70.6–84.2)	88.0 (83.6–92.4)	93.6 (90.2–97)	97.9^c^ (95.6–100)	2.9 (0.6–5.2)	2.0 (0.1–3.9)	0.0^c^ (0)

(4) *N* = 265,259,205	49.4 (43.4–55.4)	66.8^a^ (61.1–72.5)	88.8^b,c^ (84.5–93.1)	90.2 (86.6–93.3)	95.8^a^ (93.6–98.2)	97.6^c^ (95.5–99.7)	3.0 (0.9–5)	1.2 (0–2.5)	0.0^c^ (0)

(5) *N* = 218,216,0	40.8 (34.3–47.3)	63.4^a^ (57–69.8)		86.7 (82.2–91.2)	91.7 (88–95.4)		2.8 (0.6–5)	1.9 (0.1–3.7)	

(6) *N* = 0,257,253		75.5 (70.2–80.8)	92.5^b^ (89.3–95.7)		89.5 (85.8–93.2)	94.5^b^ (91.7–97.3)		1.6 (0.1–3.1)	0.8 (0–1.9)

(7) *N* = 0,171,0		55.6 (48.2–63)			79.5 (73.4–85.6)			4.1 (1–7)	

*N*: number of colonoscopies; SAR: sedation administration rate; CI: 95% confidence interval; CCR: colonoscopy completion rate; ADR: adenoma detection rate; CR: early complications rate; ^a^significance over Era 1; ^b^significance over Era 2; ^c^significance of Era 3 over Era 1.

**Table 3:** Individual endoscopist performance in cohort C.

Endoscopist *N* per Era: 1, 2, and 3	Sedation administration, % (CI)			CCR, % (CI)			ADR, % (CI)			CR, % (CI)		
	Era 1	Era 2	Era 3	Era 1	Era 2	Era 3	Era 1	Era 2	Era 3	Era 1	Era 2	Era 3
(1) *N* = 20,0, 0	80.0 (62.5–97.5)			95 (85.4–100)			25 (6–44)			0 (0)		

(2) *N* = 136,0, 0	52.0 (43.6–60.4)			93.4 (89.2–97.6)			23.5 (16.4–30.6)			2,9 (0.1–5.7)		

(3) *N* = 45,64,51	37.8 (23.6–50)	60.9^a^ (51–72.9)	72.5^c^ (60.2–84.8)	91.1 (82.8–99.4)	95.3 (90.1–100)	100^c^ (100)	31.1 (17.6–44.6)	39.1 (27.1–51.1)	38.0 (24.7–51.3)	4.4 (0–10.4)	1.6 (0–4.7)	0.0 (0)

(4) *N* = 78,115,85	46.2 (35.1–57.3)	66.1^a^ (57.4–74.8)	90.6^b,c^ (84.4–96.8)	93.6 (88.2–99)	99.1 (97.4–100)	98.8 (96.5–100)	25.6 (15.9–35.3)	36.5 (27.7–45.3)	42.4^c^ (31.9–52.9)	1.3 (0–3.8)	0.9 (0–2.6)	0.0 (0)

(5) *N* = 40,89,0	50.0 (34.5–64.0)	71.9^a^ (64.6–81.2)		92.5 (84.3–100)	94.4 (89.6–99.2)		30 (15.8–44.2)	27 (17.8–36.2)		2.5 (0–7.3)	2.0 (0–4.9)	

(6) *N* = 0,83,98		78.3 (69.4–87.2)	94.9^b^ (90.5–99.3)		96.4 (92.4–100)	96.9 (93.5–100)		22 (13.1–30.9)	27.6 (18.7–36.5)		2.4 (0–5.7)	2.1 (0–4.9)

(7) *N* = 0,51,0		62.7 (49.4–76)			86.4 (77–95.8)			25.5 (13.5–37.5)			2.5 (0–6.8)	

*N*: number of colonoscopies; SAR: sedation administration rate; CI: 95% confidence interval; CCR: colonoscopy completion rate; ADR: adenoma detection rate; CR: early complications rate; ^a^significance over Era 1; ^b^significance over Era 2; ^c^significance of Era 3 over Era 1.
